# *Mnsod1* promotes the development of *Pleurotus ostreatus* and enhances the tolerance of mycelia to heat stress

**DOI:** 10.1186/s12934-022-01878-2

**Published:** 2022-08-08

**Authors:** Ludan Hou, Zongqi Liu, Kexing Yan, Lijing Xu, Mingchang Chang, Junlong Meng

**Affiliations:** 1 College of Food Science and Engineering, Shanxi Agricultural University, 1 Mingxian South Road, Taigu, 030801 Shanxi China; 2 Shanxi Research Center for Engineering Technology of Edible Fungi, Taigu, 030801 Shanxi China; 3 Shanxi Key Laboratory of Edible Fungi for Loess Plateau, Shanxi Agricultural University, Taigu, 030801 Shanxi China

**Keywords:** *Pleurotus ostreatus*, Manganese superoxide dismutase, Overexpression, RNA interference, Development, Heat stress

## Abstract

**Background:**

Superoxide dismutases (SODs, EC 1.15.1.1) are defense proteins that can be used as sweepers to clear reactive oxygen species (ROS). They have been widely studied in the plant. Intensive research demonstrates that SOD plays an essential role in plants. However, in *Pleurotus ostreatus*, the function and regulatory pathway of SOD in the growth and development and the abiotic stress response have not been clear.

**Results:**

In this study, three MnSOD-encoding genes of the *P. ostreatus* CCMSSC00389 strain were cloned and identified. *Mnsod1*, *Mnsod2*, and *Mnsod3* were interrupted by 3, 7, and 2 introns, and encoded proteins of 204, 220, and 344 amino acids, respectively. By comparing the relative expression of three MnSOD-encoding genes in mycelia, the results showed that the gene with the highest primary expression was *Mnsod1*. Subsequently, the function of *P. ostreatus Mnsod1* was explored by overexpression (OE) and RNA interference (RNAi). The results showed that during the growth and development of *P. ostreatus*, MnSOD1 protein increased gradually from mycelia to the fruiting body, but decreased in spores. The change of *Mnsod1* transcription level was not consistent with the changing trend of MnSOD1 protein. Further studies showed that during primordia formation, the expression of *Mnsod1* gradually increased, reaching a peak at 48 h, and the transcription level was 2.05-folds compared to control. H_2_O_2_ content progressively accumulated during the formation of primordia, and its change trend was similar to that of *Mnsod1* transcription. OE-*Mnsod1*-1 and OE-*Mnsod1*-21 strains accelerated the formation of primordia. The results suggested that *Mnsod1* may participate in the formation rate of *P. ostreatus* primordium by regulating the signal molecule H_2_O_2_. In addition, OE-*Mnsod1*-1 and OE-*Mnsod1*-21 strains shortened the mycelial recovery time after heat stress and improved the tolerance of the strains to 2.5 mM and 5 mM H_2_O_2_, which showed that *Mnsod1* was involved in the response of *P. ostreatus* mycelium to heat stress.

**Conclusions:**

This study indicates that *Mnsod1* plays an active role in the formation of *P. ostreatus* primordia and the response to abiotic stress.

**Supplementary Information:**

The online version contains supplementary material available at 10.1186/s12934-022-01878-2.

## Background

Superoxide dismutases (SODs, EC 1.15.1.1) are ubiquitous in animals, plants, microorganisms, and fungi. They are a kind of metalloproteins, which usually contain iron, manganese, copper, zinc, or nickel as auxiliary groups [1]. As early as 1969, the biological function of SOD was revealed. As the first enzyme involved in the scavenging reaction of reactive oxygen species (ROS), SOD can catalyze the superoxide anion produced in organisms into oxygen (O_2_) and hydrogen peroxide (H_2_O_2_) [2]. In addition, SOD is the core of antioxidant enzymes, which can participate in the response of organisms to a variety of abiotic stresses and physiological and biochemical reactions[3]. SOD can be divided into many types due to different metal ions. Different kinds of SOD can be located in different positions in organisms [4]. For example, in plants, Cu/ZnSOD was situated in various organelles, including chloroplasts and peroxisomes, and secondly in the cytoplasm. FeSOD was mainly found in chloroplasts, and MnSOD was located in mitochondria. Then, the number of SOD isozymes coding genes varies with species [5, 6]. For example, there are 8 SOD coding genes in the *Arabidopsis* genome, while 17 SOD isozyme coding genes have been identified in *Brassica rapa* [7]. In fungi, SOD has also been cloned and studied in recent years. For example, in *Phytophthora nicotianae*, MnSOD activity was detected in mycelia, zoospores, and germinated cysts [8]. In *Phytophthora cinnamomi*, five kinds of MnSOD with a relative molecular weight of 25–100 kDa were found [9]. In *Candida albicans*, four Cu/ZnSODs and two MnSODs were identified [10, 11].

As the components of the antioxidant defense system, the biological functions of SOD have been widely studied. For example, in rice, the OE of the MnSOD coding gene of peas can enhance the tolerance of plants to drought stress [12]. In *Arabidopsis*, OE of the jojoba MnSOD coding gene can improve the drought resistance of plants [13]. In *Medicago sativa* L., the OE of *Fesod* promoted the recovery ability of plants after cold stress and reduced the secondary injury caused by ROS [14]. In addition, SOD also plays a vital role in plant growth and development. In *Arabidopsis*, after mitochondrial MnSOD was suppressed by antisense agents, the root growth of the recombinant strain was slow, and mitochondrial redox homeostasis was specifically disturbed in seedlings grown in liquid culture [15]. In fungi, research on the function of SOD has also gradually been carried out. In *Aspergillus fumigatusmmi*, the expression of *sod1* and *sod2* was the highest in conidia, and *sod3* was the highest in mycelia. Compared to other *sods*, the expression of *sod4* was weak, but the deletion of *sod4* was fatal [16]. In *Cryptococcus neoformans*, two kinds of SODs were identified, Cu/ZnSOD in cytoplasm and MnSOD in mitochondria, which were required for full virulence [17]. However, there are few reports on the function of SOD in edible mushrooms.

*Pleurotus ostreatus* is one of the most widely cultivated edible mushrooms globally, with high nutritional and medicinal value [18]. In China, the cultivation of *P. ostreatus* is mainly horticultural facilities. Compared to factory cultivation, the environmental factors in horticultural facilities, such as humidity and carbon dioxide concentration, especially temperature, are challenging to control. For example, in the mycelial growth stage, heat stress will significantly inhibit the mycelial growth rate. In the stage of fruiting body development, the high-temperature weather in summer will also affect the development of primordia and fruiting bodies [19]. This abiotic stress has seriously affected the yield of *P. ostreatus*. With the successful application of genetic transformation technology in mushrooms, the research focus has also rapidly shifted from the cultivation of mushrooms to the exploration of gene functions and regulatory pathways. The publication of many mushroom genomes has laid a foundation for the study of gene function. At present, the research on the development of the fruiting body and the stress response mechanism of mushrooms has been gradually carried out. Some genes in edible fungi have been identified. Their functions have been studied, such as cytochrome P450 [20], nicotinamide adenine dinucleotide phosphate oxidase [21], catalase [22], phenylalanine ammonia-lyase [23], and alternative oxidase [24]. Second, the function of transcription factors in the growth and development of edible mushrooms has also become the focus of research, such as primordium development defect PDD1 [25], the novel putative Zn(II)2Cys6 transcription factor LFC1 [26], the negative transcription regulator FvHmg1 [27], and the transcription factors Bri1 and Hom1 [28]. In addition, the AMP and the NO signaling pathway have been reported in mushrooms [29, 30]. Recently, the latest research results showed that mechanical injury could cause an increase in ROS in mycelia and further promote the formation of *P. ostreatus* primordia [31]. SOD is a defense line to clear ROS. Its function and regulatory mechanism in *P. ostreatus* deserve further study.

In this work, three MnSOD-encoding genes were cloned. The gene structure, phylogeny, and amino acid sequence alignment of three MnSOD isozyme-coding genes were analyzed. Then, the relative expression of three MnSOD isozyme coding genes was compared, and the coding gene with the highest expression was screened. Finally, the role of MnSOD in the development of *P. ostreatus* and heat stress was explored by constructing RNAi- and OE-recombinant strains.

## Results

### Cloning and bioinformatics analysis of MnSOD-encoding genes

**Fig. 1:**
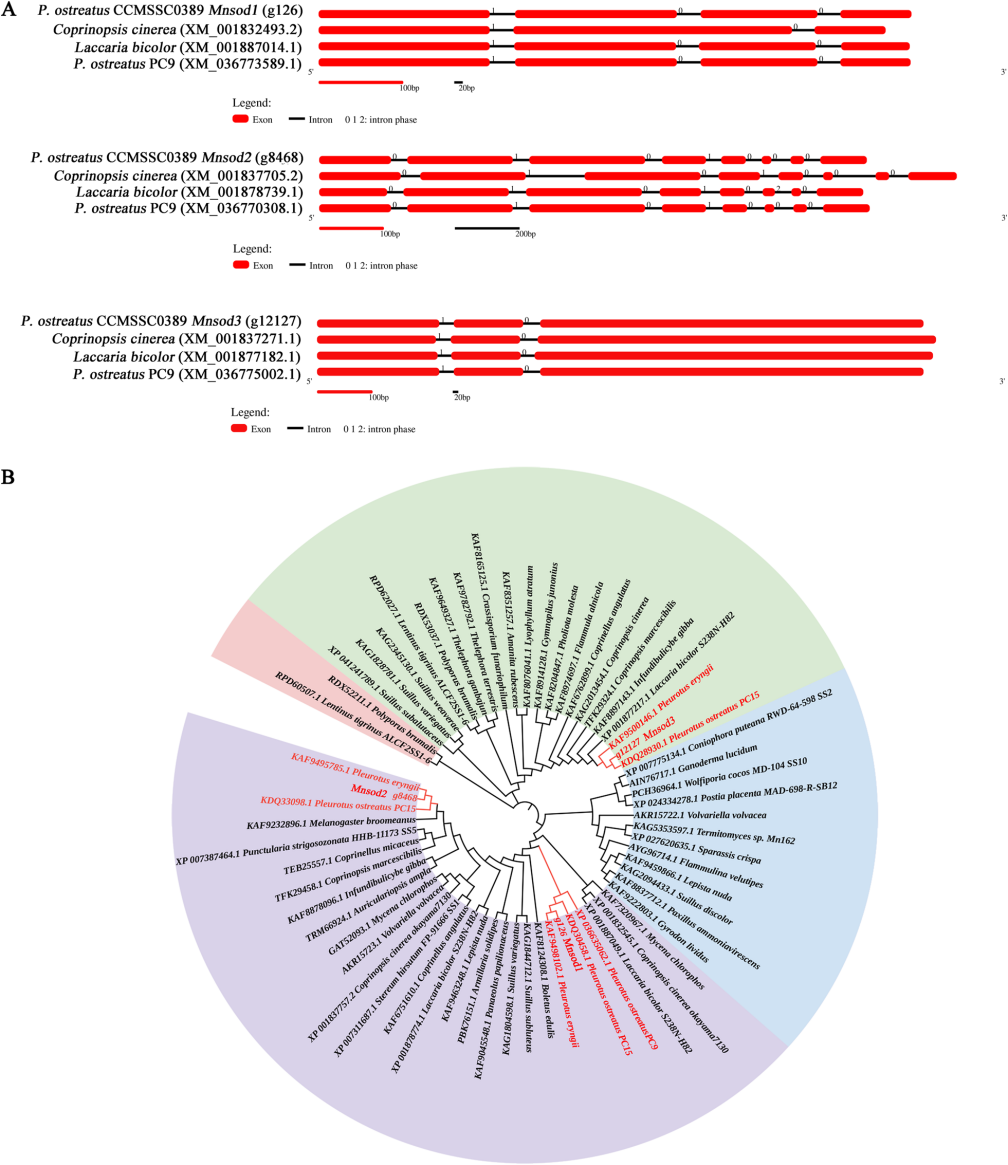
Gene structural features and relationships of fungal MnSOD. **A** Gene structures of selected MnSOD-encoding genes in *P. ostreatus* CCMSSC00389, *Coprinopsis cinerea*, *Laccaria bicolor*, and *P. ostreatus* PC9. The exons are represented by red rectangles, and the black lines connecting two exons represent introns. **B** A neighbor-joining phylogenetic tree of 63 MnSOD protein sequences from fungal species

In the genome of *P. ostreatus*, SOD includes two types: MnSOD and Gu/ZnSOD. In this study, three MnSOD coding genes were identified from the *P. ostreatus* CCMSSC00389 genome. They were named g126 (*Mnsod1*), g8468 (*Mnsod2*), and g12127 (*Mnsod3*), and their full-length cDNA sequences were 615, 663, and 1035 bp, respectively. DNA sequence analysis showed that 4 exons were interrupted by 3 introns in g126 (*Mnsod1*), 8 exons were interrupted by 7 introns in g8468 (*Mnsod2*), and 3 exons were interrupted by 2 introns in g12127 (*Mnsod3*) (Fig. 1A). Figure 1A shows that g126 (*Mnsod1*) and g8468 (*Mnsod2*) had a similar gene structure with the genes encoding *P. ostreatus* PC9 and *L. bicolor* but quite different from that of the model organism *C. cinerea*. However, the gene structure of g12127 (*Mnsod3*) was similar to that of *P. ostreatus* CCMSSC00389, *P. ostreatus* PC9, *L. bicolor*, and *C. cinerea*.

63 MnSOD protein sequences were selected to analyze the phylogenetic relationship between the MnSOD protein of *P. ostreatus* and other fungal MnSODs (Fig. 1B). The results showed that three MnSOD protein sequences were clustered into two major branches. MnSOD1 and MnSOD2 clustered in the same branch. The results indicated that g126 (MnSOD1) and g8468 (MnSOD2) were more closely related. It is speculated that MnSOD1 and MnSOD2 may have similar biological functions. MnSOD3 (g12127) was clustered in another branch, which indicated that the biological function of MnSOD3 may be different from those of MnSOD1 and MnSOD2 (Fig. 1B).

**Fig. 2:**
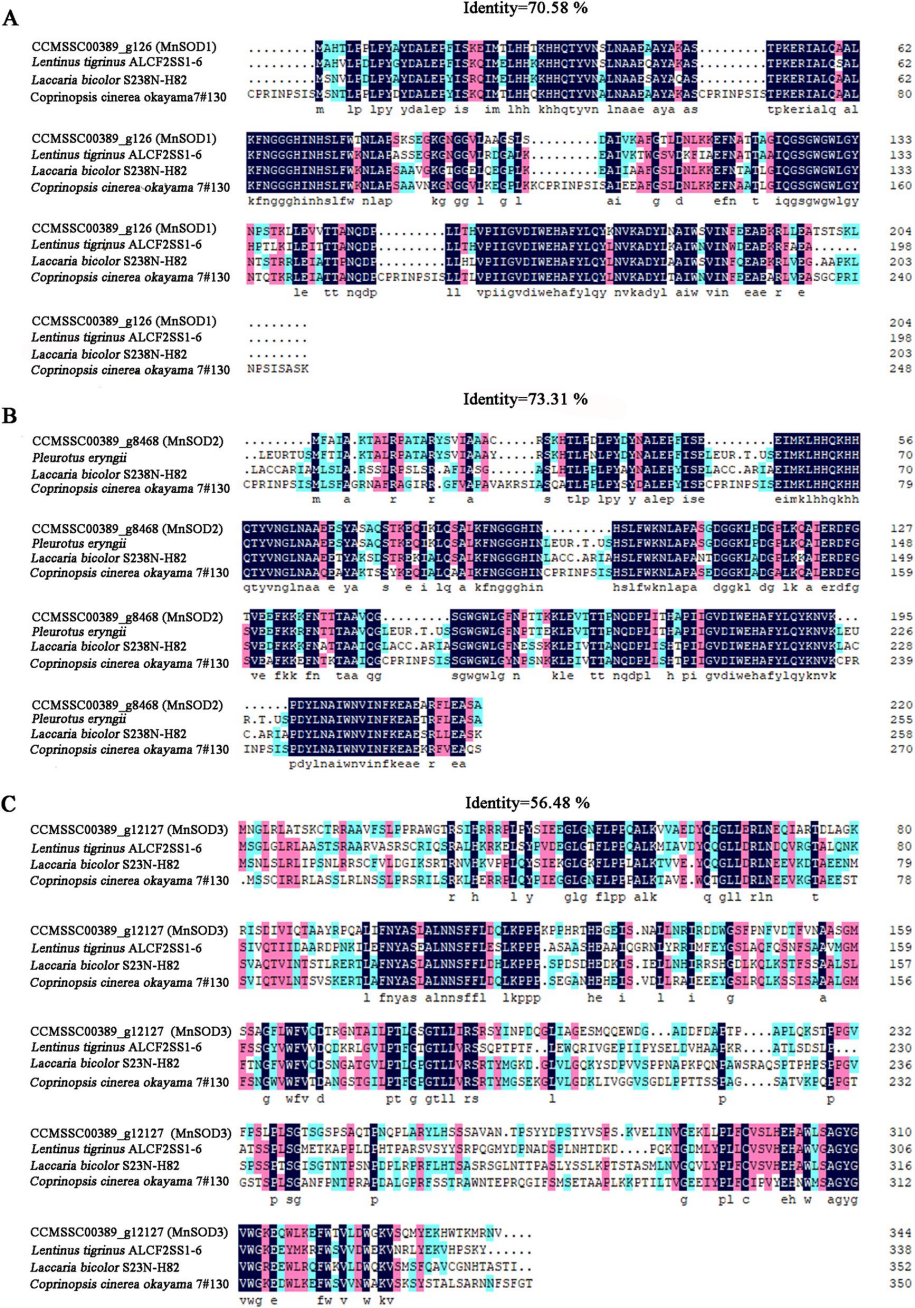
Amino acid sequence alignment of MnSODs between *P. ostreatus* and other fungi. **A** g126 (MnSOD1) of *P. ostreatus* CCMSSC00389 and other fungi (*Lentinus tigrinus* ALCF2SS1-6_RPD60507.1; *L. bicolor* S238N-H82_XP_001887049.1; *C. cinerea okayama*7#130_XP_001832545.1). **B** g8468 (MnSOD2)of *P. ostreatus* CCMSSC00389 and other fungi (*P. eryngii*_KAF9495785.1; *L. bicolor* S238N-H82_XP_001878774.1; *C. cinerea okayama* 7#130_XP_001837757.2). **C** g12127 (MnSOD3) of *P. ostreatus* CCMSSC00389 and other fungi (*L. tigrinus* ALCF2SS1-6_RPD62027.1; *L. bicolor* S238N-H82_XP_001877217.1; *C. cinerea okayama*7#130_XP_001837323.1)

Bioinformatics analysis of the *Mnsod* sequence showed that g126 (*Mnsod1*) encoded a hypothetical 204 amino acid polypeptide with a molecular weight of 22.29 kDa and a calculated isoelectric point of 6.75. The homology of MnSOD amino acid sequences in different organisms is described in Fig. 2. The results showed that MnSOD1 (g126) was highly similar to the SOD coding genes of *L. tigrinus* (RPD60507.1), *L. bicolor* (XP_001887049.1) and *C. cinerea okayama* (XP_001832545.1), and the consistency reached 70.58% (Fig. 2A). Figure 2B shows that MnSOD2 (g8468) was a polypeptide chain composed of 220-amino-acid residues, up to 73.31% consistent with the homologous proteins in *P. eryngii*, *L. bicolor*, and *C. cinerea okayama*. The online prediction results showed that the approximate molecular weight of the MnSOD2 (g8468) protein was 24.51 kDa and the isoelectric point was 7.85. The *Mnsod3* (g12127) gene encodes a 344 amino acid polypeptide, and its molecular weight and predicted isoelectric point are 38.18 kDa and 8.35, respectively. Second, MnSOD3 (g12127) was compared with the homologous proteins of *L. tigrinus* (RPD62027.1), *L. bicolor* (XP_001877217.1), and *C. cinerea okayama* (XP_001837323.1). The results showed that the identity was low (54.68%). In conclusion, MnSOD proteins are well conserved in different fungi.

To further analyze the MnSOD proteins in *P ostreatus* CCMSSC00389, the identity between MnSOD1 (g126), MnSOD2 (g8468) and MnSOD3 (g12127) was compared. The results showed that the sequence identity of MnSOD1 (g126) and MnSOD2 (g8468) was as high as 63.6%. It can be inferred that the biological functions of MnSOD1 and MnSOD2 may be similar. However, the amino acid sequences of MnSOD3 and the other two amino acid sequences had low similarity in *P. ostreatus* CCMSSC00389 (Additional file 1: Fig. S1). It is speculated that the function of MnSOD3 may be quite different from MnSOD1 and MnSOD2 in *P. ostreatus*. This result is consistent with the phylogenetic tree analysis result in Fig. 1B.

**Fig. 3:**
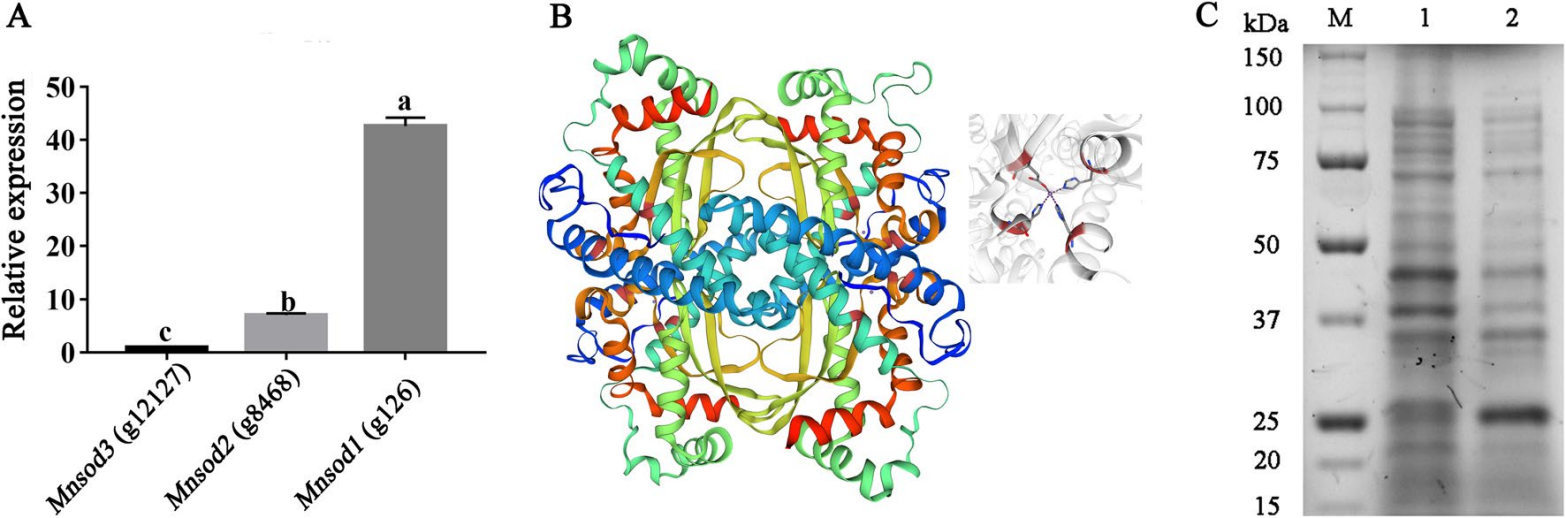
3D structural model of the MnSOD1 protein and sodium dodecyl sulfate–polyacrylamide gel electrophoresis (SDS-PAGE) gel analysis of recombinant MnSOD1. **A** Determination of the relative expression of MnSOD encoding genes. **B** 3D structural model of the MnSOD1 protein. **C** SDS-PAGE analysis of recombinant MnSOD1 protein. M, marker; 1, crude lysate without Isopropyl-β-D-thiogalactopyranoside (IPTG); 2, crude enzyme induced with IPTG

To further explore the structure and function of MnSOD in *P. ostreatus*, the relative expression levels of three MnSOD-encoding genes were compared. Figure 3A shows that the expression of the *Mnsod1* (g126) gene was significantly higher than that of the other two encoding genes. It is speculated that the *Mnsod1* (g126) gene may have more critical functions. The three-dimensional (3D) structure of MnSOD1 was predicted using 6s0d. 1 (SMTL ID) as a template (Fig. 3B). The results showed that MnSOD1 was a homologous tetrameric protein, and the amino acids at positions 27(H), 72(H), 161(D), and 165 (H) of each monomer interacted with Mn metal ions. Prokaryotic expression analysis showed that the actual molecular weight of MnSOD1 was about 25 kDa (Fig. 3C), which was consistent with the predicted result (22.29 kDa).

### Acquisition of *Mnsod1* mutant strains

**Fig. 4:**
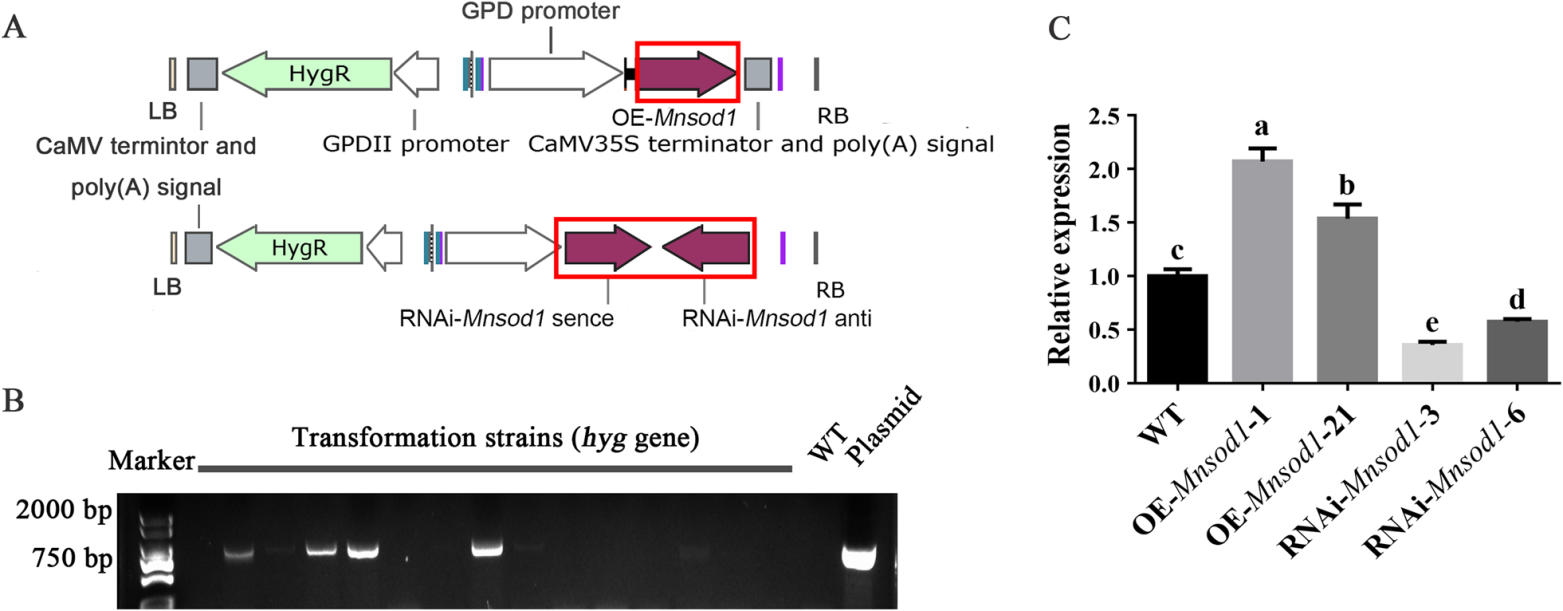
Screening and acquisition of *Mnsod1*-recombinant strains. **A** The OE-*Mnsod1* and RNAi-*Mnsod1* plasmids map. **B** Amplification of *hyg* gene in *Mnsod1* mutant strains. **C** The relative expression of the *Mnsod1* in mutant strains

In this work, the biological function of *Mnsod1* in *P. ostreatus* was explored by constructing mutant strains. Figure 4A shows the OE and RNAi plasmid map of *Mnsod1*. In the maps, the *hyg* gene was used as a screening marker for recombinant strains. The constructed OE-*Mnsod1* and RNAi-*Mnsod1* plasmids were transformed into the mycelia of *P. ostreatus* by *Agrobacterium*-mediated genetic transformation technology. First, the *hyg* gene fragment was amplified for further screening (Fig. 4B). Then, the expression of the *Mnsod1* gene in the recombinant strains was determined. Figure 4C showed that the expression of the *Mnsod1* gene in OE-*Mnsod1*-recombinant strains was significantly up-regulated compared to that in the WT strain. In contrast, the expression of the *Mnsod1* gene in RNAi-*Mnsod1* strains was significantly down-regulated. The *Mnsod1* mutant strains of *Mnsod1* were obtained for further research.

### Expression pattern of MnSOD1 in the growth and development of *P. ostreatus*

**Fig. 5:**
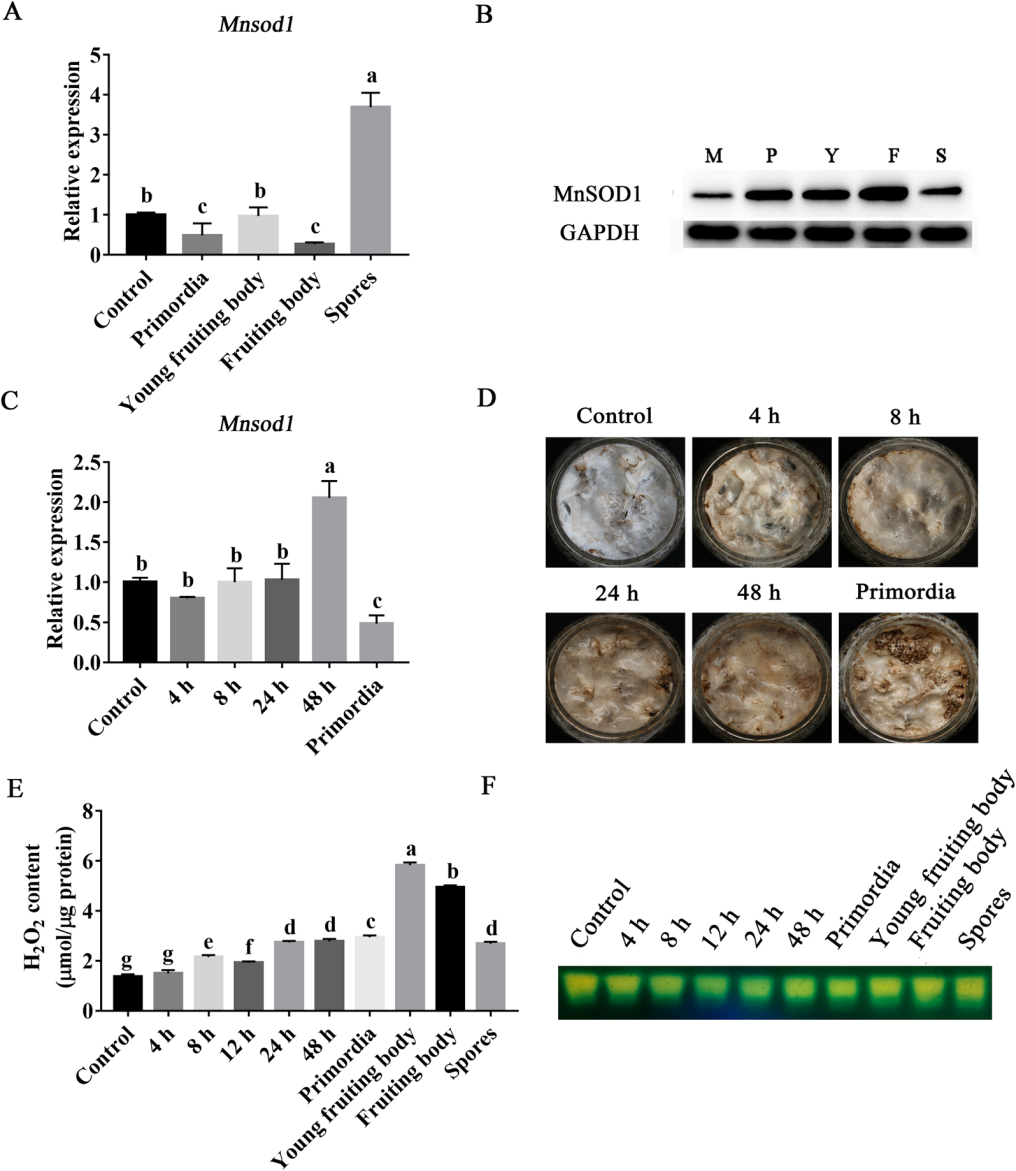
Expression pattern of MnSOD1 and changes in H_2_O_2_ content in the development of *P. ostreatus*. **A** Expression of the *Mnsod1* gene in different developmental stages. **B** Expression of MnSOD1 protein in different developmental stages. **C** Changes in the transcription of the *Mnsod1* gene during primordia formation. **D** In situ staining of H_2_O_2_ during primordia formation. **E** Changes in H_2_O_2_ content in different developmental stages. **F** Changes in CAT enzyme activity in different developmental stages. In the mushroom experiment, when the mycelium was full in the cultivation bottles, collect the mycelia as the control group

In plants, MnSOD plays an essential role in root elongation, flower formation, and mitochondrial homeostasis [15, 32]. In the development of *P. ostreatus*, the expression patterns of *Mnsod1* and MnSOD were detected to explore the function of MnSOD. The results showed that the expression of the *Mnsod1* gene in primordia decreased to 48.36% compared to that in mycelia. There was no significant difference in *Mnsod1* expression between the young fruiting body and mycelia. In the fruiting body, the expression of *Mnsod1* was down-regulated to 27.05% compared to the mycelial stage. In spores, the expression of *Mnsod1* was significantly up-regulated, which was 3.69-folds than that in mycelia (Fig. 5A). Figure 5B showed that the expression of MnSOD1 protein in mycelia, primordia, young fruiting bodies, and fruiting bodies followed a gradual increasing trend. In spores, the expression of MnSOD1 protein is significantly down-regulated. On the other hand, the expression of MnSOD1 protein was significantly up-regulated in the process of vegetative growth to reproductive growth, so it is speculated that MnSOD1 may be essential in the formation of primordia. Surprisingly, the changing trend of the transcription of the *Mnsod1* gene was not consistent with the MnSOD1 protein test results, especially in the primordial stage. The transcription of the *Mnsod1* gene was significantly down-regulated (Fig. 5A), while MnSOD1 protein was significantly accumulated (Fig. 5B). To further explore the reason, the expression of the *Mnsod1* was additionally detected in mycelia at 4 h, 8 h, 24 h, and 48 h during the process of primordia formation. Figure 5C showed that the expression of the *Mnsod1* in mycelia did not change significantly within 24 h after transfer to the mushroom room. Still, at 48 h, the expression of the *Mnsod1* gene increased significantly by 2.05-folds compared to mycelia and decreased significantly after primordia formation (Fig. 5C). Since it takes time from gene expression to protein assembly and maturation, it is speculated that the considerable accumulation of MnSOD1 protein in the primordia may be caused by the high expression of the *Mnsod1* gene in the early stage of primordia formation.

H_2_O_2_ is the product of SOD. Figure 5D shows the results of H_2_O_2_ in situ staining during the primordia formation. H_2_O_2_ content gradually accumulated during primordia formation, especially at 48 h, and noticeable browning was observed. By comparing Fig. 5D and C, it can be found that the changing trend of H_2_O_2_ content in mycelia during primordia formation is highly similar to that of the transcription of the *Mnsod1* gene. It is speculated that MnSOD1 may participate in the formation of primordia by regulating the amount of H_2_O_2_ during the growth and development of *P. ostreatus*. Subsequently, the H_2_O_2_ content in primordia formation and different developmental stages was quantitatively detected. The results showed that during the formation of primordia, H_2_O gradually accumulated (Fig. 5E), and CAT enzyme activity decreased slightly (Fig. 5F), which further promoted the accumulation of H_2_O_2_. In addition, this result is consistent with that of Fig. 5D. Second, Fig. 5E shows that the H_2_O_2_ content in the young fruiting body was significantly increased by 1.98-folds compared to that in primordia. This further proves the vital role of H_2_O_2_ in the formation of primordia. It is speculated that H_2_O_2_ may also play a vital role in differentiating primordia into fruiting bodies.

In conclusion, MnSOD1 in *P. ostreatus* may affect primordia formation and differentiation by regulating H_2_O_2_.

### *Mnsod1* is involved in primordia formation

**Fig. 6:**
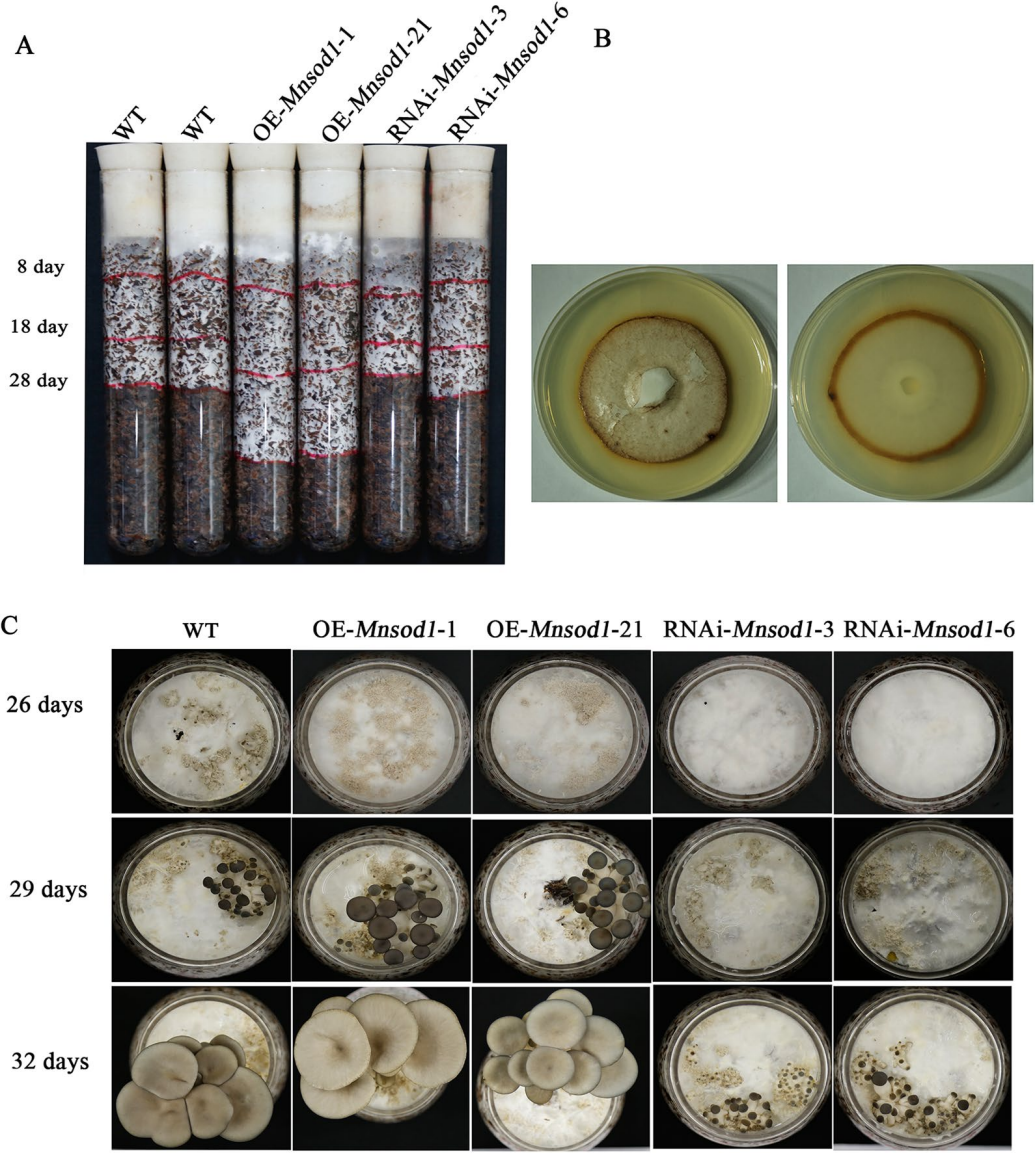
Effect of *Mnsod1* on the development of *P. ostreatus*. **A** Detection of the growth rate of *Mnsod1*-recombinant strains in cultivation substrate. **B** In situ staining of H_2_O_2_ in plate mycelia. **C** Effect of *Mnsod1*-recombinant strains on mushroom production of *P. ostreatus*

To further explore the role of MnSOD1 in the development of *P. ostreatus*, *Mnsod1* recombinant strains were constructed. First, the growth rate of the recombinant strains in the cultivation substrate was detected. The results showed that compared to the WT strain, the growth rate of OE*-Mnsod1* strains was significantly hastened. In contrast, RNAi*-Mnsod1* strains showed no significant difference (Fig. 6A). Subsequently, by in situ staining of plate mycelia, it was found that H_2_O_2_ accumulated significantly at the mycelial tip (Fig. 6B). Considering that H_2_O_2_ has the function of a signaling molecule in plant growth and development, it is speculated that the accumulation of H_2_O_2_ at the tip of plate mycelia may be related to the rapid growth of mycelia, and H_2_O_2_ may play an essential role as a signaling molecule in the process of mycelial growth. In conclusion, *Mnsod1* OE may promote mycelial growth by increasing H_2_O_2_ content. Figure 6C shows that compared to the WT strain, the OE*-Mnsod1* strains slightly promoted the rapid formation of primordia and shortened the developmental cycle of the fruiting body. In contrast, RNAi*-Mnsod1* strains can significantly prolong the primordia formation time and prolong the fruiting body development cycle. In conclusion, *Mnsod1* plays an active role in the development of *P. ostreatus*.

### *Mnsod1* participates in the regulation of the mycelial response to heat stress

**Fig. 7:**
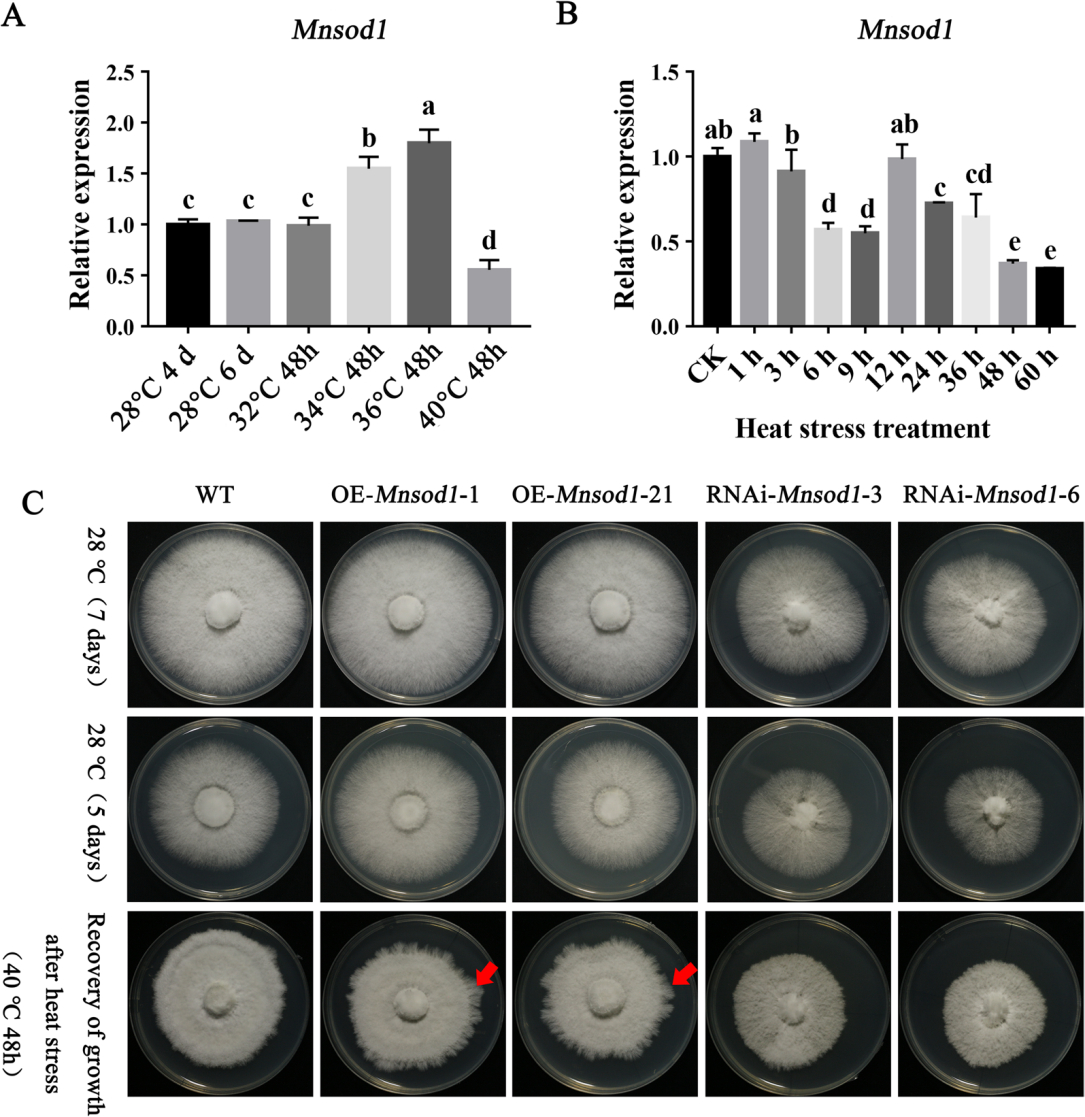
*Mnsod1* OE enhanced the tolerance of strain to heat stress. **A** Detection of *Mnsod1* expression in mycelia under different temperature stresses. **B** Detection of *Mnsod1* expression in mycelia after heat stress at 40 °C for different times. **C** Comparison of the recovery growth rate of *Mnsod1* mutant strains after heat stress

According to previous studies, MnSOD plays a crucial role in abiotic stress response [33, 34]. In this study, the function of *Mnsod1* in response to heat stress of *P. ostreatus* was preliminarily explored. Figure 7A shows the expression of *Mnsod1* in plate mycelia cultured at 28 °C for 4 days under stress treatment at 32 °C, 34 °C, 36 °C, and 4 °C for 48 h. The results showed that compared to 28 °C plate mycelia, the expression of *Mnsod1* did not change significantly after stress at 32  °C for 48 h. When the stress temperature increased to 34 °C and 36 °C, the expression of *Mnsod1* increased by 1.49-folds and 1.68-folds, respectively. It is speculated that the significant upregulation of *Mnsod1* was involved in the stress response of mycelia to heat stress at 34 °C and 36 °C. Under heat stress (40 °C 48 h), the expression of *Mnsod1* was significantly down-regulated compared to the plate mycelia cultured at 28 °C. It is speculated that the mycelia were seriously damaged by heat stress at 40 °C for 48 h. Figure 7B shows that the expression of *Mnsod1* showed a regular change trend within 36 h at 40 °C. Therefore, the mycelia can also respond to stress by regulating gene expression within 36 h of 40 °C. However, when the stress time was prolonged to 48 h and 60 h, the expression of *Mnsod1* gradually decreased, indicating that the mycelia could no longer participate in the stress response by regulating gene expression after 48 h of stress at 40 °C. Therefore, 40 °C stress for 48 h is the best treatment time for further research.

Then, the mycelia under heat stress for 48 h at 40 °C were transferred and placed at 28 °C to restore growth. Figure 7C shows that the OE of *Mnsod1* shortened the recovery time of mycelia after heat stress and promoted the recovery rate of mycelia. On the contrary, the recovery growth rate of RNAi-*Mnsod* strains after heat stress slowed down compared to that of the WT strain. In conclusion, *Mnsod1* can actively participate in the response of *P. ostreatus* to heat stress.

## Discussion

SOD is one of the most crucial defense means to scavenge free radicals. In plants, the function of SOD has been studied deeply. However, the biological role of SOD in edible fungi has not been fully elucidated. Therefore, genetic transformation technology to explore its function in edible fungi has important research significance. First, the number, structure, and species of SOD ecological coding genes are different in different species. Research reports have shown three types of SOD isozymes in potatoes [35]. In *Salvia miltiorrhiza*, 8 *sod* genes, including 3 *Cu/Znsods*, 2 *Fesods*, and 3 *Mnsods*, were identified [36]. However, in the genome of *P. ostreatus*, two types of SOD isozymes were found, namely, Cu/ZnSOD and MnSOD. *Fesod* was not annotated. In this study, three MnSOD encoding genes of *P. ostreatus* were identified. Research reports show that *Mnsod* is highly conserved in mitochondria of different plants [37]. Similar to plants, MnSOD was also highly conserved in fungi (Figs. 1A and 2). Therefore, it is speculated that MnSOD may play a similar function in different fungi. Through phylogenetic tree analysis and amino acid sequence alignment, it was found that g126 (*Mnsod1*) was more similar to g8468 (*Mnsod2*), and g12127 (*Mnsod3*) was quite different from them. Therefore, it is speculated that the functions of g126 (*Mnsod1*) and g8468 (*Mnsod2*) may be more similar in *P. ostreatus*, while g12127 (*Mnsod3*) may be different from g126 (*Mnsod1*) and g8468 (*Mnsod2*). Further analysis of g126 (*Mnsod1*) with the highest primary expression showed that MnSOD1 was assembled as a homotetramer and contained one Mn atom per tetramer. This was similar to the results in plants [38].

Recently, the regulatory mechanism of *P. ostreatus* growth and development has become the research focus. In this process, the formation of primordia is very important. In the mushroom production stage, it needs to be stimulated by low temperature and light. Previous studies have shown that the content of ROS in *P. ostreatus* mycelia increased after mechanical damage, and primordia were formed in advance [25]. In *G. lucidum*, primordia could not be formed normally after reducing the level of ROS in mycelia by RNAi [15]. The rapid formation of primordia is closely related to the increase in ROS in mycelia. H_2_O_2_ is an integral part of ROS. In plants, H_2_O_2_ is considered as an important signal molecule, which can participate in plant development and mediate various physiological processes [40], including seed germination [41], flowering [42], root development [43], stomatal aperture regulation [44] and many others. SOD is closely related to H_2_O_2_, and SOD activity has been detected in a variety of mushrooms, which have high thermal stability [39]. In this study, by constructing *Mnsod1*-recombinant strains, it was found that OE-*Mnsod1* strains showed the early formation of primordia. In contrast, the primordia in RNAi-*Mnsod1* strains were delayed. Therefore, it is speculated that the OE and RNAi of *Mnsod1* may affect the formation rate of primordia by regulating H_2_O_2_. The regulatory pathway of H_2_O_2_ in the formation of primordia and the development of *P. ostreatus* is still unknown and is worthy of further study.

**Fig. 8:**
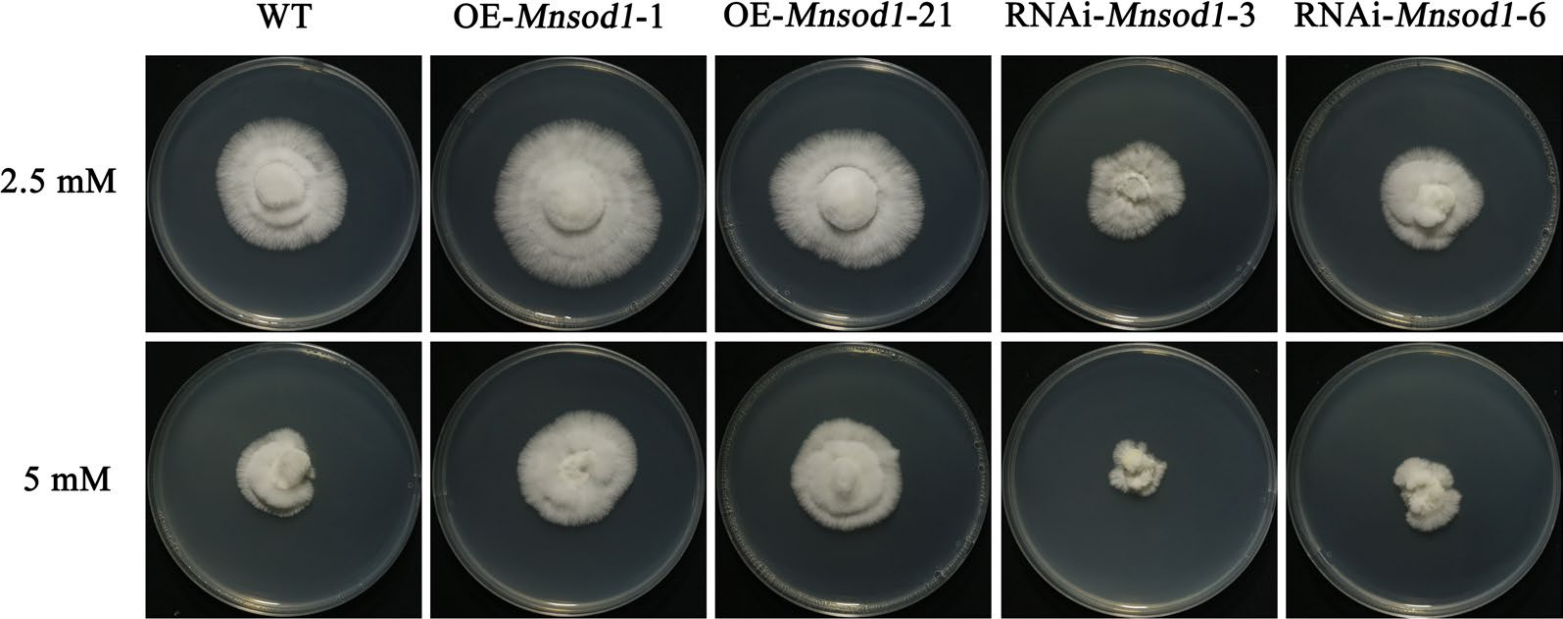
*Mnsod1* OE enhances the tolerance of mycelia to H_2_O_2_

In the cultivation and production of *P. ostreatus*, heat stress is critical abiotic stress affecting mycelial growth, fruiting body quality, and yield. Heat stress causes an outbreak of ROS in the mycelial growth stage, which destroys the integrity of the cell wall and promotes pollution by *Trichoderma asperellum* [45, 46]. SOD is an essential enzyme in the antioxidant enzyme system. Currently, many studies have confirmed that SOD can participate in the response process of plants to abiotic stress and play a positive role [47, 48]. In this study, *Mnsod1* OE can shorten the recovery time of mycelia after heat stress, accelerating the recovery growth rate. In contrast, *Mnsod1* RNAi slowed the mycelial recovery rate after heat stress. Previous studies have shown that heat stress can lead to the accumulation of H_2_O_2_ in *P. ostreatus* mycelia, and further cause oxidative damage to mycelia [25, 30]. Therefore, the exogenous addition of H_2_O_2_ was used to simulate the outbreak of H_2_O_2_ in mycelium after heat stress, which further clarified that OE-*Mnsod1* strains could improve the tolerance of mycelia to H_2_O_2_ (Fig. 8). It is speculated that the high expression of *Mnsod1* under heat stress may participate in the heat stress response pathway of *P. ostreatus* by regulating H_2_O_2_. The role of MnSOD in abiotic stress has been widely studied. In *Candida albicans*, the mutant that knocks out mitochondrial MnSOD was more sensitive to various abiotic stresses than WT cells [49]. In potatoes, RNAi of the *sod1* gene decreased SOD activity and increased the sensitivity of plants to cold stress; On the contrary, OE of the *sod1* gene enhanced the tolerance to cold [50]. In rice, *Mnsod*-recombinant lines treated with polyethylene glycol (PEG) 6000 showed reduced electrolyte leakage and enhanced resistance to methyl viologen- or PEG-induced oxidative stress compared to WT [12]. The current is similar to those in plants. In conclusion, SOD plays a positive role in the fungal stress response [51]. In addition, SOD can also be used to treat diseases [52]. Plat-derived SOD can be ingested as a diet to enhance antioxidant circulation and reduce oxidative stress [53]. Therefore, it is speculated that the edible fungus SOD has broad application prospects in the health care and medical industries.

## Conclusions

Three MnSOD-encoding genes were cloned and analyzed. The MnSOD encoding gene *Mnsod1* had the largest primary expression by qPCR analysis. It was further observed that the *Mnsod1* gene changed regularly in different developmental stages of *P. ostreatus*, and the MnSOD1 protein increased gradually from mycelia to fruiting bodies. In addition, *Mnsod1* was involved in the response of mycelia to heat stress. Then, by constructing the recombinant strains of *Mnsod1*, it was confirmed that *Mnsod1* OE could promote the rapid formation of primordia in the process of growth and development and promote the recovery of the mycelial growth rate after heat stress. This study preliminarily confirmed that *Mnsod1* not only plays an active role in the development of *P. ostreatus*, but also participates in the response of mycelia to abiotic stress. This result will provide a basis for further exploring the function and regulatory pathway of MnSOD in fungi.

## Materials and methods

### Strains

The *P. ostreatus* CCMSSC00389 strain was provided by the Center for Mushroom Spawn Standards and Control of China and preserved in the Mushroom Germplasm Resource Center. The *Agrobacterium tumefaciens* (*A. tumefaciens*) GV3101 strain was preserved in the laboratory. The OE-*Mnsod1* and RNAi-*Mnsod1* plasmids were constructed with the original plasmids also kept in the same laboratory. The vector pET28a (Novagen, Inc., Madison, WI, USA) was used for prokaryotic expression experiment.

### Cloning and analysis of MnSOD-encoding genes

The three MnSOD-encoding genes were identified by keyword search from *P. ostreatus* PC15 strain genome database. Subsequently, the nucleotide sequences were used to design primers to amplify MnSOD coding genes. Amino acid multiple sequence alignment and phylogenetic tree construction were carried out by DNAMAN software and MEGA software, respectively. The molecular weight and the isoelectric point were predicted by ProtParam. The 3D structure was created by Swiss-Model, and the *Mnsod* structures were predicted by Gene Structure Display Server 2.0.

### Purification of recombinant MnSOD1 protein

The plasmid construction and recombinant MnSOD1 protein purification methods of the prokaryotic expression experiment were the same as our previous research methods [23]. Briefly, the constructed plasmid was transformed into *E. coli* BL21 (DE3) for induction expression. The experiment was divided into two groups: the first with no added IPTG as the uninduced group, whereas the second enclosed 1mM IPTG to induce MnSOD1 protein expression. After that, SDS-PAGE was used to analyze the fractions.

### Construction of OE-*Mnsod1* and RNAi‑*Mnsod1* plasmids

The original pCAMBIA1300 vector was modified in a previous study [23]. According to that method, the OE-*Mnsod1* and RNAi-*Mnsod1* plasmids were constructed. Finally, the OE-*Mnsod1* and RNAi-*Mnsod1* plasmids were introduced into *A. tumefaciens* GV3101. The primers used in this study are shown in Additional file 1: Table S1.

### Mushroom production experiment

The *P. ostreatus* CCMSSC00389 strain was cultured three times. Subsequently, the cultivation bottles (cottonseed shell medium) were prepared, and the mycelia were inoculated on cottonseed shell medium and cultured at 25 °C. When the mycelia were complete, the mushroom production experiment was carried out. In the mushroom production experiment, cultivation bottles were divided into equal groups, with 10 cultivation bottles in each group. Five groups were used to collect samples at different developmental stages. The other groups were used to collect samples during primordia formation, including 0 h, 4 h, 8 h, 12 h, 24 h, 48 h, and primordia. Then, the samples were used to detect the expression pattern of the *Mnsod1* and MnSOD1 protein, H_2_O_2_ content, and CAT enzyme activity. The mushroom production method of *Mnsod1* recombinant strains was consistent with the above-described process.

### Western blot analysis

The expression pattern of MnSOD1 in the development of *P. ostreatus* was detected by Western blotting according to the method of a previous study [22]. Briefly, the total protein was extracted from different samples (mycelia, primordia, young fruiting bodies, fruiting bodies, spores), and the protein concentration was detrected by Detergent Compatible Bradford Protein Quantification Kit (Vazyme, Nanjing, China). Then, the equal amount of total protein (20 μg) of different samples was separated in 12% (w/v) SDS-PAGE gel. After electrophoresis, the protein was transferred to polyvinylidene difluoride membranes. Finally, MnSOD1 antibodies (GenScript, Nanjing, China) were used for Western blot analysis, with glyceraldehyde 3-phosphate dehydrogenase (GAPDH) antibodies (GenScript, Nanjing, China) as a reference.

### Determination of H_2_O_2_ content

The H_2_O_2_ content in different samples was determined by an H_2_O_2_ Quantitative Assay Kit (Sangon Biotech, Shanghai, China).

### Staining to detect the H_2_O_2_

The changes of H_2_O_2_ during the formation of *P. ostreatus* primordia were detected according to previous research methods [54]. To detect H_2_O_2_, the sample was stained with 1 mg/mL 3,3N-diaminobenzidine in 10 mM potassium phosphate (pH 6.5) for 30 min at room temperature and then washed with distilled water. Then, the staining was observed after 7 h of culture in a mushroom growing room.

### Quantitative PCR (qPCR)

The expression of MnSOD-encoding genes in differently treated samples was detected by qPCR. The total RNA in different samples was extracted using the E.Z.N.A. Plant RNA Kit (Omega Bio-Tek, Norcross, GA, USA), and cDNA was synthesized using the HiScript II 1st Strand cDNA Synthesis Kit (Vazyme, Nanjing, China) for qPCR analysis as previously described [22]. In this study, the *β-actin* gene was used as a reference. The relative expression of genes was calculated according to the 2^−△△CT^ method.

### Heat stress treatment

The WT strain was inoculated on PDA plates, cultured in the dark at 28 °C for 5 days, and then transferred to stress treatment at different temperatures (32 °C, 34 °C, 36 °C, 40 °C) for 48 h. After receiving the sample, it was used to detect the changes of *Mnsod1* expression. On this basis, the expression pattern of *Mnsod1* was detected after heat stress (40 °C) for different times (1 h, 3 h, 6 h, 9 h, 12 h, 24 h, 36 h, 48 h, 60 h).

### Determination of the recovery growth rate of mycelium after heat stress

According to previous research methods [55], the activated strains of WT, OE-*Mnsod1*-1, OE-*Mnsod1*-21, RNAi-*Mnsod1*-3, and RNAi-*Mnsod1*-6 were inoculated in PDA plates, cultured in the dark at 28 °C for 5 days, treated at 40 °C for 48 h, and then recovered to grow at 28 °C. Mycelial recovery and growth were observed and photographed.

### H_2_O_2_ susceptibility assay

According to previous research methods, the sensitivity of WT, OE, and RNAi-*Mnsod1* strains to H_2_O_2_ was determined [23]. 5 mm pellets of test strains were inoculated onto PDA plates containing different concentrations of H_2_O_2_ (0 mM, 2.5 mM, 5 mM). After 7 days of incubation at 28 °C, the diameter of strains was measured and photographed.

### Statistical analysis

Graphpad prism 6 was used for the statistical analysis in this study. The values are means ± SEs. The different letters among the samples denote significant differences (*P* < 0.05 according to Duncan’s test).

## Supplementary Information

**Additional file 1: Table S1.** Primers used in this study. **Fig. S1.** Partial amino acid sequence alignment of three MnSODs of *P. ostreatus* CCMSSC00389. **A** g126 (MnSOD1) vs g8468 (MnSOD2). **B** g126 (MnSOD1) vs g12127 (MnSOD3) . **C** g8468 (MnSOD2) vs g12127 (MnSOD3)

## Data Availability

All data generated or analysed during this study are included in this published article and the additional files.

## Abbreviations

*P. ostreatus*: *Pleurotus ostreatus*
SOD: Superoxide dismutase
ROS: Reactive oxygen species
OE: Overexpression
RNAi: RNA interference
O2: Oxygen
H2O2: Hydrogen peroxide
PEG: Polyethylene glycol
3D: Three-dimensional
IPTG: Isopropyl-β-d-thiogalactopyranoside
SDS-PAGE: Sodium dodecyl sulfate–polyacrylamide gel electrophoresis
GAPDH: Glyceraldehyde 3-phosphate dehydrogenase
qPCR: Quantitative PCR
