# Supplementary material for: Mnsod1 promotes the development of Pleurotus ostreatus and enhances the tolerance of mycelia to heat stress
Source: Microb Cell Fact. 2022 Aug 8;21:155. doi: 10.1186/s12934-022-01878-2 (PMC9358896; doi:10.1186/s12934-022-01878-2)
Supplement: Supplementary file 1 — Additional file 1: Table S1. Primers used in this study. Fig. S1. Partial amino acid sequence alignment of three MnSODs of P. ostreatus CCMSSC00389. A g126 (MnSOD1) vs g8468 (MnSOD2). B g126 (MnSOD1) vs g12127 (MnSOD3) . C g8468 (MnSOD2) vs g12127 (MnSOD3) [file 12934_2022_1878_MOESM1_ESM.docx]

**Additional file 1**

**Table S1.** Primers used in this study.

| Primer | Sequence (5′→3′) | Note |
| --- | --- | --- |
| *Mnsod1*_F | ATGGCCCACACACTCCCACC | gDNA and cDNA fragment cloning |
| *Mnsod1*_R | TTACAGCTTGGAAGTGGAGGTGGC |  |
| *Mnsod2*_F | ATGTTCGCCATCGCCAAAACCGC |  |
| *Mnsod2*_R | CTACGCAGAGGCTTCGAGGAAACGAGCT |  |
| *Mnsod3*_F | ATGAATGGCCTCCGTTTAGCCACCTCAA |  |
| *Mnsod3*_R | CTAAACGTTTCGCATCTTGGTCCAGTGCTTCT |  |
| *Mnsod1-*PE-F | TCGCGGATCCGAATTCATGGCCCACACACTCCCAC | Construction of PE plasmid |
| *Mnsod1-*PE-R | TGCTCGAGTGCGGCCGCTTACAGCTTGGAAGTGGAGGTGG |  |
| OE-*Mnsod1*-F | GGTCAAAGTTACTAGTATGGCCCACACACTCCCAC | Construction of OE and RNAi plasmids |
| OE-*Mnsod1*-R | CAATTCTAGAGGGCCCTTACAGCTTGGAAGTGGAGGTGG |  |
| RNAi-*Mnsod1*-Sence-F | CCATCTCCTCAGATCTATGGCCCACACACTCCCAC |  |
| RNAi-*Mnsod1*-Sence-R | TAAGCTCTAAACTAGTTTACAGCTTGGAAGTGGAGGTGG |  |
| RNAi-*Mnsod1*-Anti-F | CAAGCTGTAAACTAGTCGGCTTCCTCGAAGTTGATGAC |  |
| RNAi-*Mnsod1*-Anti-R | CAATTCTAGAGGGCCCATGGCCCACACACTCCCAC |  |
| *hyg*-F | CGACAGATCCGGTCGGCATCTACTCTATTTCTT | Detection of transformants |
| *hyg*-R | TCTCGTGCTTTCAGCTTCGATGTAGGAGGG |  |
| *β-actin*-F | GCGATGAACAATAGCAGGG | Endogenous control |
| *β-actin*-R | GCTGGTATCCACGAGACAAC |  |
| *Mnsod1*-qF | ACACGAAGCATCATCAGACCTA | qPCR |
| *Mnsod1*-qR | GAAGAGCGAGTGGTTGATATGG |  |
| *Mnsod2*-qF | GTTCTCCTAGCAGCGAAGA |  |
| *Mnsod2*-qR | CATTCCCCGTTTTAAGTGAC |  |
| *Mnsod3*-qF | TTGAACGAGACTTTGGCACC |  |
| *Mnsod3*-qR | ATGATCGGCGCGTGAGTTATC |  |


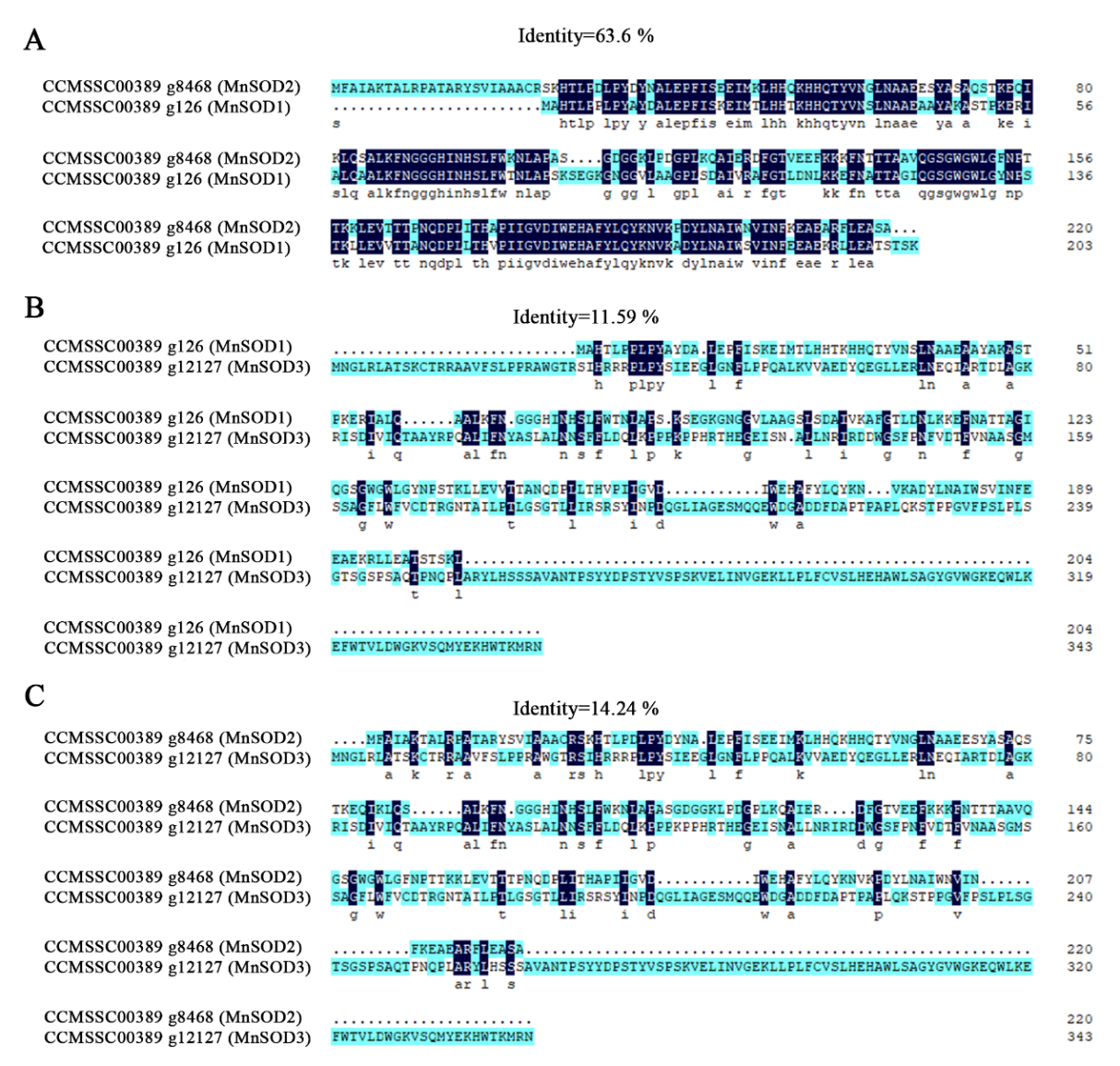


**Fig. S1.** Partial amino acid sequence alignment of three MnSODs of *P. ostreatus* CCMSSC00389. A. g126 (MnSOD1) vs g8468 (MnSOD2); B. g126 (MnSOD1) vs g12127 (MnSOD3); C. g8468 (MnSOD2) vs g12127 (MnSOD3).
